# Characterizing serotonin expression throughout bovine mammary gland developmental stages and its relationship with 17β-estradiol at puberty

**DOI:** 10.1371/journal.pone.0319914

**Published:** 2025-03-25

**Authors:** Sena L. Field, Lisa M. Arendt, Laura L. Hernandez, Jimena Laporta

**Affiliations:** 1 Department of Animal and Dairy Sciences, University of Wisconsin-Madison, Madison, Wisconsin, United States of America; 2 Department of Comparative Biosciences, University of Wisconsin-Madison, Madison, Wisconsin, United States of America; Tokat Gaziosmanpaşa University: Tokat Gaziosmanpasa Universitesi, TÜRKIYE

## Abstract

Serotonin acts in an autocrine/paracrine manner within the mammary epithelium regulating cell homeostasis during lactation and cell turnover during involution after milk stasis. However, the presence and role of mammary serotonin during the pubertal developmental stage is unknown in the bovine. Here, we characterized the serotonin receptor profile and serotonin immunolocalization in bovine mammary tissue at eight developmental stages (i.e., birth, weaning, puberty, six months gestation, early lactation, mid-lactation, early dry and late dry, n = 6/stage). Further, we investigated the effects of 5-HTP (serotonin precursor), 17β-estradiol (E_2_), and ICI 182780 (ERα antagonist) either alone or in various combinations (i.e., 5-HTP +  E_2_, 5-HTP + ICI, E_2_ + ICI or 5-HTP + E_2_ +  ICI) on cultured bovine mammary epithelial cells (MAC-T). Serotonin receptor gene expression is highly dynamic throughout mammary development, particularly highly expressed in the puberty stage expressing 12 out of the 13 serotonin receptors evaluated (*5-HTR1A*, *-1B*, *-1D*, *-1F*, *-2A, -2B, -2C, -3B, -4, -5a, -6,* and *-7*), relative to the birth stage. Following a 24-hour incubation, all treatments except ICI increased MAC-T cell proliferation. Incubation with 5-HTP +  ICI resulted in a downregulation of *ESR1*, *ESR2*, *GPER1* and *AREG,* relative to CON. Incubation with 5-HTP and E_2_ alone downregulated the expression of *TPH1*, *5-HTR1A* and *5-HTR1B*, relative to CON. Overall, our data indicates serotonin is present in the juvenile developing mammary tissue and the expression of various receptors is observed suggesting an active involvement at this early stage. Additionally, serotonin might indirectly regulate mammary epithelial cell proliferation alone and concurrently with E_2_ during puberty through the modulation of E_2_ signaling genes and *5-HTR1A* and *-1B*.

## Introduction

Serotonin (5-hydroxytryptamine) is a multifunctional monoamine derived from the amino acid L-tryptophan. Tryptophan hydroxylase 1 (TPH1, rate liming step enzyme) converts L-tryptophan to 5-Hydroxy-L-tryptophan, which is then converted to serotonin by the aromatic amino acid decarboxylase (AADC, ubiquitous enzyme). Serotonin regulates a myriad of physiological processes due to the presence of more than 15 evolutionary conserved serotonin receptor subtypes (5-HTR1- 5-HTR7) and various isoforms throughout the body [1].

Serotonin is considered a “local regulator” in the mammary epithelium due to its autocrine/paracrine mode of action [2]. Serotonin acts as a homeostatic regulator of lactation where *TPH1* activation is reliant upon alveolar distension following secretory activation [3]. Additionally, the presence of five serotonin receptor isoforms (*5-HTR1B, -2A, -2B, -4* and *-7*) transcripts were identified in lactating bovine mammary tissue and primary bovine mammary epithelial cells [4]. During mammary involution, serotonin signals through 5-HTR7 in mammary epithelial cells (MEC) to regulate alveolar milk volume, milk protein expression, and mammary epithelial cell tight junctions [5,6]. The bovine mammary gland serotonergic system has been investigated in lactation and involution [7,8]. Yet, the expression of serotonin and its receptors in the pre-pubertal and pubertal mammary gland is unknown.

Mammary gland growth and development begins during embryogenesis from the ectoderm, giving rise to a rudimentary ductal tree. At birth, the mammary gland primarily consists of the fat pad and a smaller portion of parenchyma (i.e., epithelial cells) [9]. During post-natal development through puberty in humans, mice and cows, the mammary gland undergoes dramatic allometric growth consisting of ductal branching and elongation under the hormonal control of ovarian steroids. Specifically, epithelial cell expansion is driven in part by 17β-estradiol (E_2_) signaling through its receptor estrogen receptor alpha (ERα) and progesterone signaling through its receptor progesterone (P_4_) receptor (PR) by a paracrine mechanism [10]. Amphiregulin, an epidermal growth factor ligand downstream of ERα signaling, is augmented by ERα activation and is required to induce mammary epithelial cell proliferation, terminal end bud formation and ductal elongation [11]. Additionally, ovariectomy (removing the source of P_4_ and E_2_ in heifers) has been shown to blunt mammary growth, further supporting their crucial role [12,13].

The relationship between estrogens and the serotonergic system in the brain is well established, however, the associations between the two in the peripheral system are beginning to be elucidated [14]. In human trophoblasts, serotonin signaling through *5-HTR2A* increases aromatase (CYP19A1, rate liming enzyme) producing E_2_ [15]. Further, *in vivo* studies in rats and macaques have shown that E_2_ regulates *TPH1* expression and inhibits the serotonin transporter (SERT) [14]. Additionally, virgin mice administered fluoxetine showed a decrease in circulating E_2_, uterine weight and altered ovarian morphology [16]. Overall, data suggests an interaction between serotonin and E_2_ in placental and reproductive function, although there is no literature documenting the serotonin E_2_ interaction in the mammary gland.

The objectives of this study were to determine the presence of serotonin and its receptors throughout various key stages of bovine mammary gland development (i.e., birth, weaning, puberty, mid-gestation, lactation, and the dry period) and to investigate the potential relationship between serotonin and E_2_ in regulating the expression of genes related to E_2_ signaling and mammary proliferation in MAC-T cells. We hypothesized serotonin receptor expression and presence is dynamic throughout mammary gland development and may play a role as a regulatory molecule during pubertal mammary epithelial cell proliferation by acting synergistically with E_2_.

## Materials and Methods

### Animals and experimental design

This experiment was approved by the Institutional Animal Care and Use Committee at the University of Florida and University of Wisconsin-Madison. The animal studies were reviewed and approved by the Institutional Animal Care and Use Committees at the University of Florida (#201910599) and the University of Wisconsin-Madison (#A006377). Mammary tissue was collected from Holstein calves, heifers and cows (n = 48, n = 6 biological replicates/developmental stage) during birth (4.6 ± 2.3 hr old), weaning (62.9 ± 1.5 d old), puberty (365 ± 7.1 d old), six months gestation (623.5 ± 11.6 d old), early lactation (14 ± 0.0 days in Milk [DIM], pregnant, 1.3 ± 0.8 lactation year [yr]), mid lactation (84 ± 0.0 DIM, pregnant, 1.3 ± 0.8 lactation yr), early dry period (d4, pregnant and non-lactating, 4 ± 0.0 d dry) and late dry period (d36 pregnant and non-lactating, 35.8 ± 2.3 d dry). Birth and weaning calves differed and were euthanized to obtain mammary parenchyma. Heifers at puberty and six months gestation differed and were biopsied instead to avoid euthanizing an adult animal. Cows during early and mid-lactation stages were the same, and cows at early and late dry periods were the same, but different from lactation cows. Similarly, all these were biopsied to obtain mammary tissue to avoid euthanizing an adult animal. Heifers at birth and weaning were euthanized to collect the whole mammary gland and dissect the parenchyma from the fat pad tissue portions, as previously described [17]. Briefly, calves after birth and weaning were euthanized via captive bolt stunning (renders the calf immediately unconscious to alleviate suffering) followed by exsanguination. After mammary dissection, a 0.5 × 0.5 cm^2^ portion of parenchyma was collected. Mammary biopsies were performed on heifers and cows at puberty, six months gestation, early lactation, mid lactation, early dry and late dry periods as described in [18] Click or tap here to enter text. Immediately after collection, the tissue was snap frozen in liquid nitrogen and stored in −80°C until RNA isolation. For immunofluorescence staining, tissue was placed in 10% neutral buffered formalin for 20 h, then transferred to PBS and stored at 4°C until paraffin embedding.

### Cell culture

Bovine mammary epithelial cells (MAC-T) were utilized for this experiment. MAC-T cells incubated at 37°C and 5% CO_2_ were plated at a seeding density of 70,000 cells/well in a 12 well plate or 30,000 cells/well in a 24 well plate, sustained in proliferation media (Phenol-red free DMEM, 10% Charcoal Stripped FBS, 10 μg/ml insulin and 1:100 antibiotic/antimycotic) and reached confluency 24 hrs later. Once confluent, new proliferation media and the following treatments were added: 500 μM 5-HTP (Sigma-Aldrich, #H9772), 1nM 17β-estradiol dissolved in DMSO (E_2_, Sigma-Aldrich, #3301), 100 nM ICI 182780 dissolved in DMSO (Med Chem Express, #HY-13636), 5-HTP + E_2_, 5-HTP + ICI, E_2_ + ICI, 5-HTP + E_2_ + ICI or DMSO (control) was added. After 24 hrs of treatment, cells were harvested for RNA isolation or entered the cell proliferation assay. Each plate contained 2 wells (i.e., replicates) of each treatment, and the experiment was repeated 3 times.

### Mammary tissue and MAC-T gene expression

Total RNA was extracted from 60 mg of mammary tissue or two combined wells of MAC-T cells using TRIzol (ThermoFisher, #15596026) per manufacturer’s instructions. RNA concentration and 260/280 absorbance were measured using Implen Nanophotometer. RNA (1 or 0.5 μg) was reverse transcribed with iScript Reverse Transcription Supermix (Bio-Rad, Hercules, CA; #1708841) and diluted (1:4 or 1:2) in molecular grade water for tissue and MAC-T cells, respectively. Quantitative real time- PCR was performed using the CFX96 Real-Time PCR detection System (Bio-Rad). Cycle protocol and reaction mixtures were performed as previously described by our group [19]. The geometric mean of three (*UXT*, *KRT8* and *CYPA*) and two (*KRT8* and *CYPA)* housekeeping genes was used to normalize gene expression for mammary tissue and MAC-T cells, respectively. We evaluated the expression of genes related to serotonin signaling (*5HTR-1A, -1B, -1D, -1F, -2A, -2B, -2C, -3A, -3B, -4, -5A, -6,* and *-7*), synthesis (*TPH1*) and recycling (*SERT*) in mammary tissue. In MAC-T cells, we evaluated the expression of genes related to E_2_ signaling (*ESR1, ESR2, AREG, GPER1*) and serotonin synthesis (*TPH1*) and receptors (*5-HTR1A, -1B* and *-7).* A gene was determined not expressed if four out of the six samples within a stage exhibited a cycle threshold value greater than 39.9 (i.e., undetectable expression). The genes *5-HTR-1A, -2C, -3A* and -6 were not detected during the birth stage. Bovine hypothalamus RNA (Zyagen #BR-204) was used as a positive control for mammary tissue and confirmed expression of all serotonin receptors. A table of primer sequences used can be found in [19] for serotonin related genes and S1 Table for E_2_ signaling genes. Using Primer3 software with sequences obtained from GenBank (http://www.ncbi.nlm.nih.gov/), all primer sequences were designed at an optimal annealing temperature of 60°C and to span exon-exon junctions, to diminish the potential of amplifying genomic DNA. All primer pairs displayed melting curves with a single peak, indicative of a pure, single amplicon, confirming the specificity of the primers. All efficiencies (10 ^(−1/slop)^ – 1 × 100]) ranged between 85% and 110%, with an R² value >  0.94. Last, primer binding specificity was tested in silico against the target genome or transcriptome to avoid off-target amplification.

### Mammary tissue immunostaining

Mammary tissue was processed using the Leica ASP300S Tissue Processor encompassing a series of washes including Pen-Fix (Epredia, #22-050-316) for 1:45 hr at 40°C, followed by a series of washes with 95% ethanol, 100% ethanol, ethanol/xylene (50/50), xylene at 40°C totaling a 13:20 hr process. Mammary tissues were then transferred to the Sakura Tissue-Tek TEC 5 EMA-1 embedding station and embedded using PureAffin X (Cancer Diagnostics, #EEPAR3).

Mammary gland paraffin blocks (birth, weaning, puberty, six months gestation, early dry and late dry n = 6/stage; early lactation, mid-lactation n = 4/stage) were sectioned in 5 μm sections on glass slides using a microtome (Thermofisher). Slides were incubated at 60°C for 30 min, deparaffinized in xylene, and rehydrated in a graded series of ethanol washes (100%, 95%, 70%). Slides were then incubated with serotonin primary antibody (Immunostar, #20080, 1:200) on a rocking plate overnight at 4°C in a light protected box. The following day, slides were incubated with secondary antibody Alexa Fluor 555 Goat Anti-Rabbit (Thermo Fisher, #A21429, 1:250) for 1 hr at 25°C in a light protected box. Nuclei were visualized using 4′,6′-diamidino-2-phenylindole (DAPI) EverBrite Mounting Medium (Biotium, #23002). The Keyence microscope (BZ-X800) was used to visualize tissue microstructure and detect fluorescence to capture five photomicrographs in one tissue section (40× magnification) per cow sample to quantify serotonin intensity. Intensity (i.e., brightness of the specified channel) in the the BZ-X800 Analyzer software is defined as the value of multiplying the area channel pixels by the mean brightness before extraction, denoted as an accurate method to acquire the amount of fluorescence expression observed. Fluorescence exposure time for all 20× images was at 1/6 seconds (s) for serotonin and 1.0 s DAPI and 40× images were captured at 1/3.5 s for serotonin and 1.2 s DAPI.

Estrogen receptor alpha (ERα) was stained in birth and puberty mammary gland sections. Briefly, 5 µm sections were treated by Citra Steam (Biogenex, #HK086-9K) for 30 min, followed by background Sniper (Biocare Medical, #BS966M) for 15 min to reduce unspecific background staining. Sections were incubated with mouse monoclonal ERα primary antibody (1:100, Santa Cruz Biotechnology #C-311sc787) for 60 min and stain was visualized using Mach 2 Mouse HRP polymer (Biocare Medical, #MHRP520L). Using the Keyence BZ-X800 microscope, four 40× magnification photomicrographs per tissue section per animal were taken to quantify ERα positive cell counts. Cells-stained brown are labeled as ERα- positive and purple cells labeled as ERα-negative.

### Cell proliferation assay

Cell proliferation was quantified in a 24-well plate using the CyQUANT Direct Cell Proliferation Assay (Invitrogen, ThermoFisher, #C35011) according to manufacturers’ instructions. Briefly, immediately following completion of treatment incubation, the detection reagent was prepared and supplemented to the cells immediately and incubated at 37°C for 1 hr. Four microphotographs were captured per well (4× magnification) with the BZ-X800 Keyence microscope to quantify cell proliferation number. Fields were selected at random avoiding overlapping and edges of the well. The BZ-X800 Analyzer software was employed to quantify cell proliferation number using the hybrid cell count tool.

### Statistical analysis

Data were analyzed by ANOVA using the MIXED procedure of SAS v. 9.4 (SAS Institute Inc, Cary, NC). Data are presented as the least squares means ± standard error of the mean (LSM ± SEM), unless otherwise stated. Mammary tissue gene expression data is presented as the model estimates (∆∆Ct, relative expression). The model for mammary tissue gene expression and serotonin immunostaining included fixed effects of treatment (birth, weaning, puberty, six months gestation, early lactation, mid-lactation, early dry and late dry period) with statistical differences tested by Dunnett and computed as all stages relative to birth. MAC-T gene expression data is presented as the model estimates (∆∆Ct, relative expression) and the model included treatment (5-HTP, β- Estradiol (E_2_), ICI 182780 (ERα Antagonist), 5-HTP + E_2_, 5-HTP + ICI, E_2_ + ICI, 5-HTP + E_2_ + ICI and DMSO (control) and block (1, 2, 3) with statistical differences compared to DMSO (control/basal) expression. Immunostaining of ERα counts was analyzed using the PROC GLIMMIX procedure in SAS. Statistical significance was declared at *P* ≤  0.05 and tendencies at 0.05 <  *P* ≤  0.10.

## Results

### Serotonin receptor expression at various developmental stages of the mammary gland

**Fig 1:**
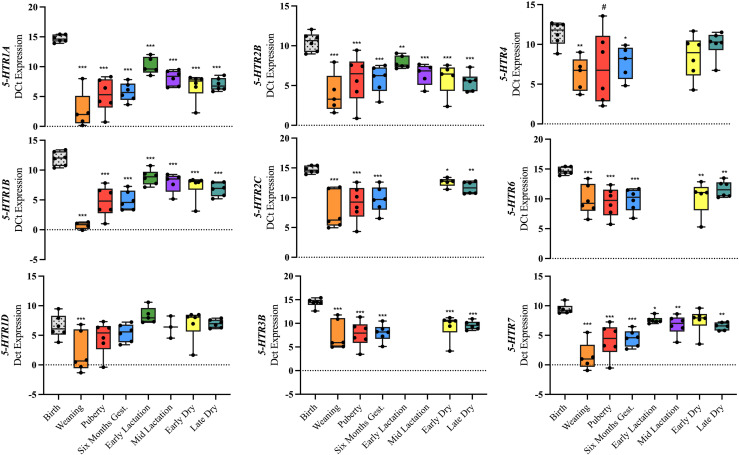
Relative mRNA expression in mammary tissue at weaning, puberty, six months gestation, early lactation, mid-lactation, early dry and late dry period of Holstein cows. Mammary tissue expression of genes related to serotonin signaling (*5HTR-1A, -1B, -1D, -2B, -2C, -3B, -4, -6,* and *-7*) were measured. Data are presented as ∆ Ct values in box and whisker plots displaying the median, first and third quartiles, as well as minimum and maximum values with individual data points. Significance declared at (*) ****P**** ≤  0.05, (**) ****P**** ≤  0.001 (***) ****P**** ≤  0.0001 and (#) denotes a statistical tendency at 0.05 <  ****P**** ≤  0.10.

To validate the expression of serotonin receptor subtypes in the mammary tissue, bovine hypothalamus was used as a positive control. We employed real time- qPCR to quantify the expression profile of 13 serotonin receptor subtypes in mammary tissue at birth, weaning, puberty, six months gestation, early lactation, mid lactation, early dry and late dry life stages. Tryptophan hydroxylase 1 (*TPH1*) and *SERT* were expressed at all mammary gland stages (S2 Fig). The serotonin receptor 1 family (i.e., *5-HTR1A, -1B, -1D, -1F*) was expressed in all 8 mammary gland stages, except *5-HTR1A,* which was not expressed at birth (Fig 1). Serotonin receptors *-1A* and *-1B* were upregulated in all mammary stages, relative to birth (*P ≤ * 0.001). The *5-HTR2B* was upregulated in all stages, relative to birth (*P* <  0.003), and *5-HTR2C* was not expressed at the early and mid-lactation stages, however, it was upregulated at weaning, puberty, six months gestation, early dry and late dry compared to birth (*P ≤ * 0.02). Serotonin receptor *-3B,* an ion-gated channel, was not expressed at birth, early lactation, or mid-lactation. However, *5-HTR3B,* was expressed and upregulated in puberty, weaning, six months gestation, early and late dry period, relative to birth (*P ≤ * 0.0001). The *5-HTR4* was not expressed at early or mid-lactation but was upregulated at weaning and six months gestation (*P ≤ * 0.02) and tended to be upregulated at puberty, relative to birth (*P* =  0.07). The *5-HTR5a* was expressed and upregulated in all stages, except early lactation, relative to birth (S1 Fig, *P ≤ * 0.004). The *5-HTR6* was not expressed at birth, early lactation, or mid-lactation but was upregulated at weaning, puberty, six months gestation, early dry, and late dry mammary tissue (*P ≤ * 0.002). Finally, *5-HTR7* was expressed and upregulated in all mammary stages, except the early dry stage, relative to birth (*P ≤ * 0.02).

### Serotonin immunostaining at various developmental stages of the mammary gland

**Fig 2:**
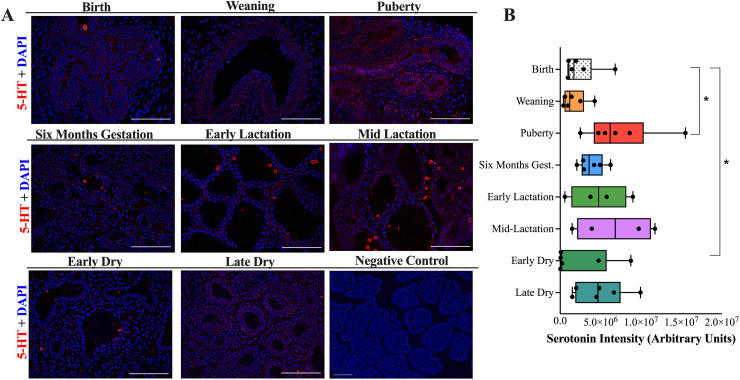
Immunofluorescence staining of (A) serotonin (5-HT) at 40X in mammary tissue at birth, weaning, puberty, six months gestation, early lactation, mid-lactation, early dry and late dry period of Holstein cows to quantify (B) serotonin intensity Scale bar =  100 μM. Negative control captured at 20X magnification. Data are presented as box and whisker plots displaying the median, first and third quartiles, as well as minimum and maximum values with individual data points. Significance declared at (*) ****P**** ≤  0.05.

The puberty stage had an increase in serotonin intensity, compared to birth (Fig 2A–B, P =  0.04). Additionally, the early dry stage had a reduction in mammary serotonin intensity, compared to birth (Fig 2A–B, P =  0.05).

### Estrogen receptor alpha mammary tissue immunostaining and MAC-T cell proliferation

**Fig 3:**
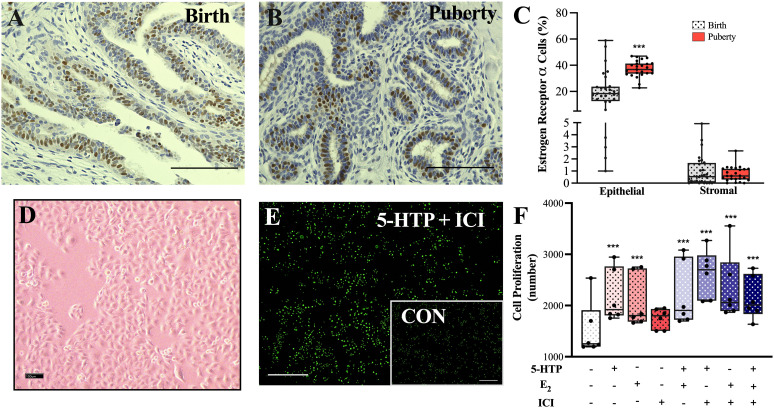
Immunohistochemistry staining estrogen receptor alpha (ER α, positive cells stained brown) in (**A**) birth and (**B**) puberty bovine mammary glands to (**C**) quantify ERα cells in the epithelial and stromal regions. Brightfield image at 10X capturing (**D**) bovine mammary epithelial cells (MAC-T) cultured in proliferation media at confluency. MAC-T cells were cultured for 24 hr at 37°C in proliferation media with one of the following treatments: 500 μM 5-HTP, 1nM β- Estradiol, 100 nM ICI 182780 (ERα Antagonist), 5-HTP +  E_2_, 5-HTP +  ICI, E_2_ +  ICI, 5-HTP +  E_2_ +  ICI or DMSO (control). (E) Representative fluorescent images at 4X capturing proliferating cells (stained green) using the cyQUANT direct cell proliferation assay to quantify (F) cell proliferation number in MAC-T cells following a 24 hr incubation of treatments. Scale bar (A–D) =  100 μM and (E) =  600 μM. Data are presented as box and whisker plots displaying the median, first and third quartiles, as well as minimum and maximum values with individual data points. Significance declared at (***) *P* ≤  0.0001 and (#) denotes a statistical tendency at 0.05 <  *P* ≤  0.10.

Estrogen receptor alpha immunostaining was greater in epithelial cells in the puberty stage mammary tissue, compared to birth (Fig 3C, P <  0.0001). Following a 24 hr incubation, all treatments except ICI increased MAC-T cell proliferation, relative to CON (Fig 3D–E, P *≤ * 0.0008).

### MAC-T cell gene expression

**Fig 4:**
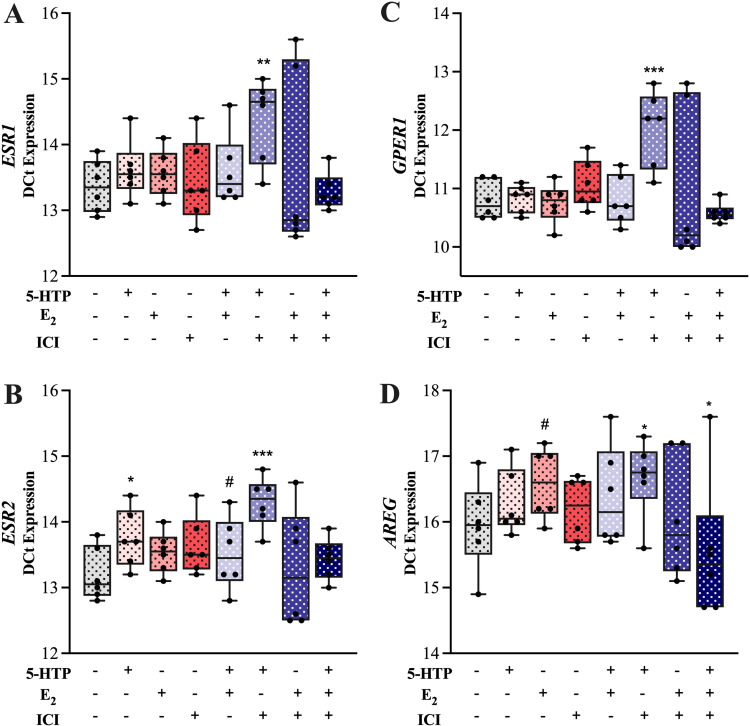
Estrogen signaling genes (*ESR1*, *ESR2, GPER1* and *AREG*) relative mRNA expression of bovine mammary epithelial cells (MAC-T) following a 24 hr incubation at 37°C in proliferation media with one of the following treatments: 500 μM 5-HTP, 1nM β- Estradiol, 100 nM ICI 182780 (ERα Antagonist), 5-HTP +  E_2_, 5-HTP +  ICI, E_2_ +  ICI, 5-HTP +  E_2_ +  ICI or DMSO (control). Data are presented as ∆ Ct values in box and whisker plots displaying the median, first and third quartiles, as well as minimum and maximum values with individual data points. Significance declared at (*) *P* ≤  0.05, (**) *P* ≤  0.001 (***) *P* ≤  0.0001 and (#) denotes a statistical tendency at 0.05 <  *P* ≤  0.10.

**Fig 5:**
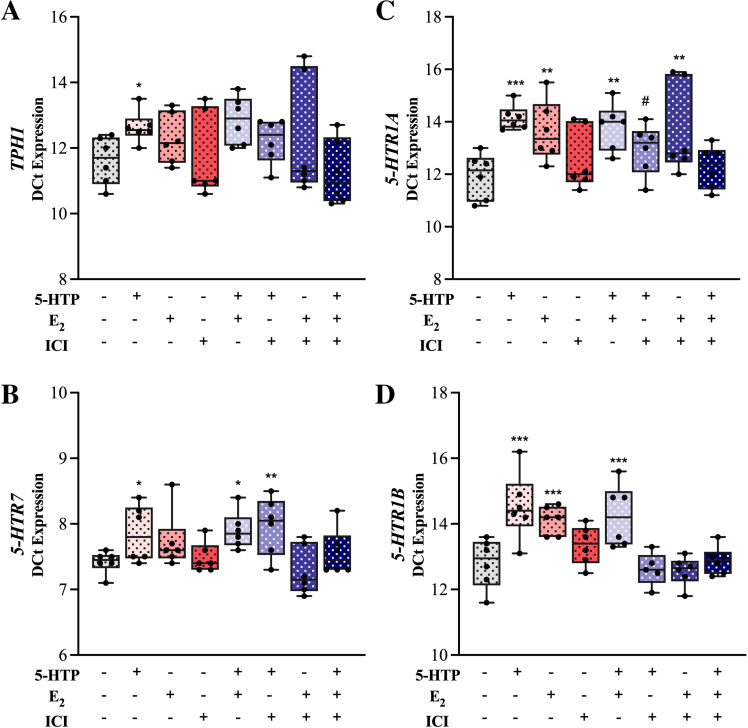
Serotonin synthesis and signaling genes (*TPH1, 5-HTR7, 5-HTR1A, 5-HTR1B*) relative mRNA expression of bovine mammary epithelial cells (MAC-T) following a 24 hr incubation at 37°C in proliferation media with one of the following treatments: 500 μM 5-HTP, 1nM β- Estradiol, 100 nM ICI 182780 (ERα Antagonist), 5-HTP +  E_2_, 5-HTP +  ICI, E_2_ +  ICI, 5-HTP +  E_2_ +  ICI or DMSO (control). Data are presented as ∆ Ct values in box and whisker plots displaying the median, first and third quartiles, as well as minimum and maximum values with individual data points. Significance declared at (*) *P* ≤  0.05, (**) *P* ≤  0.001 (***) *P* ≤  0.0001 and (#) denotes a statistical tendency at 0.05 <  *P* ≤  0.10.

To further understand the underlying molecular mechanisms, we examined the effects of 5-HTP, E_2_, ICI (ERα antagonist), 5-HTP + E_2_, 5-HTP + ICI, E_2_ + ICI, 5-HTP + E_2_ + ICI and CON (DMSO only) treatment on the MAC-T cell expression of genes related to E_2_ signaling, serotonin synthesis and signaling. (Fig 4, 5). Estrogen receptor alpha (*ESR1*) and *GPER1* were downregulated by 5-HTP +  ICI treatment, relative to CON (*P ≤ * 0.05, Fig 4A, C). Estrogen receptor beta (*ESR2*) was downregulated by 5-HTP alone and 5-HTP +  ICI and tended to be downregulated by ICI (*P ≤ * 0.08, Fig 4B). Amphiregulin (*AREG*) was downregulated by 5-HTP +  ICI and tended to be downregulated by E_2_, relative to CON (*P ≤ * 0.08, Fig 4D). Additionally, *AREG* was upregulated by 5-HTP +  E_2_ +  ICI, relative to CON (*P* =  0.02, Fig 4D). Tryptophan hydroxylase 1 (*TPH1*) was downregulated by 5-HTP and 5-HT +  E_2_ (*P ≤ * 0.05, Fig 5A). Serotonin receptor 7 (5-HTR7) was downregulated by 5-HTP, 5-HTP +  E_2_ and 5-HTP +  ICI, relative to CON (*P ≤ * 0.02, Fig 5B). Serotonin receptor 1A (*5-HTR1A)* was downregulated by 5-HTP, E_2_, 5-HTP +  E_2_, E_2_ +  ICI (*P ≤ * 0.0004) and tended to be downregulated by 5-HTP +  ICI (P =  0.08, Fig 5C). Serotonin receptor 1B (*5-HTR1B)* was downregulated by 5-HTP, E_2_ and 5-HTP +  E_2_, relative to CON. (*P ≤ * 0.0001, Fig 5D).

## Discussion

The serotonergic system plays an important role in the mammary gland during lactation and involution in the mature dairy cow [3,4,20] however, the presence of serotonin and its receptors in the mammary gland at juvenile developmental stages is unknown. Serotonin receptors activate various intracellular signaling cascades by initiating the downstream G-protein receptor alpha proteins (i.e., G_s,_ G_q/11_, G_i/o_), thus indicating a potential role for serotonin in the regulation of numerous physiological pathways within a single tissue. Therefore, delineating the serotonin receptor expression profile throughout all key mammary gland developmental stages is necessary to understand the intricate signaling pathways associated with mammary homeostasis. In the present study, we determined the presence of serotonin in the juvenile, pubertal, and mature lactating and non-lactating mammary glands, and via an *in vitro* cell culture approach, we investigated the potential interaction of E_2_ and serotonin in the mammary epithelium. We report for the first time the presence and expression pattern of serotonin and its receptors throughout bovine mammary gland developmental stages. Additionally, we begin to explore potential interactions between E_2_ and serotonin signaling in the mammary gland driving cell proliferation.

The serotonin receptor expression profile was previously characterized in pregnant lactating and non-lactating bovine mammary tissue and non-pregnant bovine mammary epithelial cells. Specifically, the serotonin receptor subtypes detected were *5-HTR-1B*, *-2A*, *-2B, -4* and *-7* by real time -qPCR, *in situ hybridization* and immunohistochemistry [4,21]. In the present study, we quantified the mRNA expression of *TPH1*, *SERT* and thirteen serotonin receptors (*5-HTR-1A, -1B, -1D, -1F, -2A, -2B, -2C, -3A, -3B, -4, -5a, -6,* and *-7*) in eight mammary gland developmental stages at birth, weaning, puberty, six months gestation, early lactation, mid lactation, early dry and late dry period. At early and mid-lactating stages, serotonin receptor subtypes detected were *5-HTR-1A, -1B, -1D, -1F, -2A, -2B, -5a* and *-7* and were upregulated relative to birth. To our knowledge, this is the first study to report the expression of *5-HTR-1A, -1D, -1F* and *-5a* in lactating bovine mammary tissue. The discrepancy with previous reports could be due in part to variations in animal age and stage of pregnancy at the time of biopsy. Selective receptor antagonists for 5-HTR-1A, -2B and -7 in primary bovine mammary epithelial cells upregulated the mRNA expression of α-lactalbumin and β-casein, suggesting a role for these receptors in regulating lactation through the modulation of milk protein transcripts [4]. Further, *5-HTR1B* was reported to be associated with a significant increase in milk production in Chinese Holsteins [20]. However, more research is necessary to delineate the roles *5-HTR-1A, -1D, -1F* and *-5a* partake in the lactating mammary gland.

In dairy cows, the dry period (i.e., non-lactating phase) is when the mammary gland undergoes extensive remodeling of mammary epithelial cells (MEC) between two consecutive lactations. The early dry period involves active involution of the gland which is characterized by an increase in apoptosis and autophagy of MEC [22]. Prior to parturition, the late dry period is characterized by an increase in MEC cell proliferation in preparation for lactogenesis. At early and late dry stages, all serotonin receptors, except 5-HTR3A, are expressed and upregulated, relative to birth. In comparison to the lactating mammary tissue, *5-HTR2C*, *-3B*, *-4* and *-6* are expressed which could suggest a role for these receptors in epithelial cell turnover occurring during the dry period. In late-lactation pregnant dairy cows, intramammary 5-HTP infusions prior to milk stasis resulted in increased mammary tissue cleaved caspase-3 staining (i.e., cell death marker) during the early dry period and an increase in mammary Ki67 staining (i.e., cell proliferation marker) during the late dry period preceding the onset of lactation [8]. These data suggests serotonin signals in the mammary gland during the dry period potentially regulating cellular turnover, although the mechanism of action is unclear. Additionally, the role of *5-HTR7* during mammary gland involution is well-recognized as the main regulator of tight junction status between MEC [5]. It is possible that the numerous other serotonin receptors present in the non-lactating mammary gland might be playing a role in mammary homeostasis. Downstream targets for these receptors need to be determined to delineate the specific subtype roles in pathways associated with cell turnover during involution.

To our knowledge, this is the first study to characterize the profile of serotonin receptors in the juvenile mammary gland. At birth, *5-HTR-1A, -2C, -3A* and -6 were not expressed. The weaning stage expressed all serotonin receptors and were upregulated, relative to birth. Additionally, at the puberty stage, all receptors except *5-HTR3A* were expressed and upregulated. The complexity of serotonin’s autocrine/paracrine signaling through the vast range of receptors expressed in the juvenile mammary gland suggests serotonin may regulate vital physiological processes during development.

Given the dynamic serotonin receptor expression throughout mammary development, we sought to determine whether mammary gland serotonin content was also dynamic throughout these mammary stages. Remarkably, the pubertal mammary gland had the greatest concentration of mammary gland serotonin, however, it is important to note the large variation between animals likely due to the small sample size. The increased expression of mammary serotonin and ERα immunostaining at puberty relative to birth was further investigated with a MAC-T cell culture model to explore the proliferative capacity and gene expression alterations of 5-HTP and E_2_ pathways cultured in isolation and concurrently. At the onset of puberty, ovarian-derived circulating E_2_ drastically rises activating ERα in a paracrine mechanism within the mammary gland [23]. The cellular and molecular mechanisms of mammary ductal development mediated by E_2_ and P_4_ are well-understood [10], although the interaction of serotonin and E_2_ in the pubertal mammary gland is unknown.

Studies have reported serotonin and E_2_ elicit proliferative effects within the mammary epithelium when supplemented in isolation, although a synergistic relationship has not been previously identified. Specifically, lactating mice administered intraperitoneal injections of 5-HTP increased Ki67 (i.e., cell proliferation marker) positive cells within the mammary gland [24]. Further, Ki67 and Cyclin D1 co-localize with ERα in rhesus monkey mammary epithelial cells [25]. In the present study, addition of 5-HTP and E_2_ alone in the media increased MAC-T cell proliferation. However, the individual effects on proliferation were not blocked by ICI as evidenced by a continuous increase in proliferation. This data is in agreeance with the current literature, whereby E_2_ effects on cell proliferation are not solely mediated through ERα. Instead, E_2_-induced proliferation is reliant on the presence of ERα in mammary stromal cells, not epithelial cells [26]. Literature suggests E_2_ mediates mammary epithelial proliferation indirectly through the regulation of hepatocyte growth factor in mammary stromal cells [27].

Incubation of 5-HTP and E_2_ together enhances MAC-T cell proliferation, although a synergistic effect did not occur as we initially expected. A plausible explanation might be that the downstream targets of 5-HTP and E_2_ inhibit one another, or they stimulate the same downstream target and that is limiting within the cell. Further investigation is necessary to delineate the downstream stimulatory and/or inhibitory transcription factors and mechanisms. The ERα antagonist, ICI, alone does not alter MAC-T cell proliferation, which might be due to the lack of E_2_ present in the media. However, when 5-HTP is supplemented with ICI, an increase in proliferation is evident. ICI 182780 is known to have agonistic effects on GPER [28], therefore, potential off target effects might exist between GPER and 5-HTR signaling due to both belonging to the G-protein coupled receptor family. Finally, the addition of E_2_, ICI and 5-HTP increased proliferation with no additive effect, suggesting ERα presence is not necessary for MAC-T proliferation. Overall, serotonin and E_2_ increase MAC-T cell proliferation with no synergistic effect, and when combined with ICI, does not attenuate this increase.

We further explore the expression of genes related to E_2_ and serotonin signaling to uncover a potential interaction in the context of mammary proliferation. 17β- estradiol elicits its effects by signaling through nuclear receptors (i.e., ERα and ERβ) and a transmembrane receptor (i.e., GPER). Upon ligand binding, the activated receptor dimerizes to interact with estrogen response elements (ERE) located at the regulatory region of target genes which activate transcription factors to regulate gene transcription positively or negatively [29]. Particular attention has been focused on the relationship between E_2_ and 5-HTR1A in various cell types. In COS-1 cells, E_2_ signaling through ERα regulates 5-HTR1A by synergistically activating the transcription factor, NFKB [30]. In the rat hippocampus, E_2_ signals through ERα to activate 5-HTR1A and phosphorylate Protein Kinase A and C [31]. Additionally, mouse macrophages secrete proinflammatory mediators in response to the synergistic activation of phosphorylated kinases by E_2_ and serotonin signaling via ERβ and GPER [32]. In agreeance with the literature, our study revealed 5-HTP, E_2_ and 5-HTP + E_2_ considerably downregulated the expression of *5-HTR1B,* and interestingly, when ICI was introduced, the effect was lost. This suggests estrogen regulates *5-HTR1B* expression in MAC-T cells through ERα. The 5-HTP + ICI treatment downregulated the expression of estrogen signaling genes *ESR1*, *ESR2*, *GPER1* and *AREG*. This novel finding suggests that serotonin communicates with estrogen receptors and their intracellular regulator (i.e., AREG), even in the absence of E_2_ in the media. Indeed, 5-HTP + ICI downregulated *AREG* expression, however, as E_2_ is added to the media, an upregulation occurs, which could potentially suggest ERβ interacts with serotonin and E_2_ in the modulation of *AREG*. Additionally, *TPH1* expression was downregulated by 5-HTP + E_2._ Research has shown estrogens modulate the expression of TPH1 in the raphe nucleus, although the exact implications are unknown [14]. Further, the 5-HTP treatment downregulated the expression of *ESR2.* In the central nervous system, research suggests estradiol regulates serotonin neuro-transmission associated with depression in women, however, research lacks an understanding of the relationship between the two molecules in the peripheral system [33].

## Conclusions

This is the first study to characterize the serotonin receptor profile in eight different developmental stages, including the juvenile bovine mammary gland. Serotonin receptor gene expression changes dynamically throughout mammary development, with the juvenile gland exhibiting greater expression and serotonin abundance compared to mature stages. The pubertal mammary gland has the greatest quantity of serotonin when compared to birth. *In vitro,* 5-HTP and E_2_ increased MAC-T cell proliferation, although the effects were not synergistic as hypothesized. Additionally, 5-HTP and E_2_ alone or combined (5-HTP +  E_2_) downregulated *5-HTR1B* by potentially indirectly signaling via ERα. Treatment of MAC-T with E_2_ downregulated the expression of TPH1, suggesting estradiol regulates serotonin synthesis through the transcriptional modulation of its rate limiting enzyme. This data lays the groundwork for future studies aimed at further unraveling the E_2_-serotonin interaction and mode of action in the pubertal mammary gland to promote MEC proliferation at this critical life stage for udder growth and development.

## Supporting information

S1 Table Primer sequences utilized for real time PCR analysis of genes involved in estrogen signaling in bovine mammary epithelial (MAC-T) cell line. All primer sequences were designed to span exon-exon junctions, to minimize the potential of amplifying genomic DNA, using Primer3 software with sequences obtained from GenBank (http://www.ncbi.nlm.nih.gov/). All primer pairs displayed melting curves with a single peak, indicative of a pure, single amplicon, confirmed the specificity of the primers. All efficiencies (10 ^(-1/slop)^ – 1 x 100]) ranged between 85% and 110%, with an R² value >  0.94. Last, primer binding specificity was tested in silico against the target genome or transcriptome to avoid off-target amplification.
(DOCX)

S1 Figure Delta Ct (DCt) expression in mammary tissue at weaning, puberty, six months gestation, early lactation, mid-lactation, early dry and late dry period of Holstein cows. Mammary tissue expression of genes related to serotonin synthesis (TPH1), uptake (SERT) and signaling (5-HTR5a). Black dots represent individual data points. Bars are presented as LSM ±  SEM. Significance declared at (*) *P* ≤  0.05, (**) *P* ≤  0.001 (***) *P* ≤  0.0001 and (#) denotes a statistical tendency at 0.05 <  *P* ≤  0.10.
(DOCX)

S1 file (XLSX)
