# Supplementary material for: Characterizing serotonin expression throughout bovine mammary gland developmental stages and its relationship with 17β-estradiol at puberty
Source: PLoS One. 2025 Mar 25;20(3):e0319914. doi: 10.1371/journal.pone.0319914 (PMC11936267; doi:10.1371/journal.pone.0319914)
Supplement: S1 Table — All primer sequences were designed to span exon-exon junctions, to minimize the potential of amplifying genomic DNA, using Primer3 software with sequences obtained from GenBank (http://www.ncbi.nlm.nih.gov/). All primer pairs displayed melting curves with a single peak, indicative of a pure, single amplicon, confirmed the specificity of the primers. All efficiencies (10 (-1/slop) – 1 x 100]) ranged between 85% and 110%, with an R² value > 0.94. Last, primer binding specificity was tested in silico against the target genome or transcriptome to avoid off-target amplification. (DOCX) [file pone.0319914.s001.docx]

**S1 Table**

Primer sequences utilized for real time PCR analysis of genes involved in estrogen signaling in bovine mammary epithelial (MAC-T) cell line. All primer sequences were designed to span exon-exon junctions, to minimize the potential of amplifying genomic DNA, using Primer3 software with sequences obtained from GenBank (http://www.ncbi.nlm.nih.gov/). All primer pairs displayed melting curves with a single peak, indicative of a pure, single amplicon, confirmed the specificity of the primers.

| **Gene** | **Forward Primer (5' --> 3')** | | **Reverse Primer (3' --> 5')** | |  |
| --- | --- | --- | --- | --- | --- |
| AREG | CCTGACATCCGTGTGTGGTT | | AGCCAAAGATGAGGAGCGAC | | |
| ESR1 | CAGGCACATGAGCAACAAAG | | TCCAGCAGCAGGTCGTAGAG | | |
| ESR2 | TCACGTCAGGCACGCCAGTAAC | | CACCAGGTTGCGCTCAGACCC | | |
| GPER1 | TTCCGCGAGAAGATGACCATCC | | TAGTACCGCTCGTGCAGGTTGA | | |
| K8 | GATGAACCGGAACATCAACC |  | GCCTGACATCCTTAACAGC |  |  |
