# Supplementary material for: Characterizing serotonin expression throughout bovine mammary gland developmental stages and its relationship with 17β-estradiol at puberty
Source: PLoS One. 2025 Mar 25;20(3):e0319914. doi: 10.1371/journal.pone.0319914 (PMC11936267; doi:10.1371/journal.pone.0319914)

**S1 Figure**

Delta Ct (DCt) expression in mammary tissue at birth, weaning, puberty, six months gestation, early lactation, mid-lactation, early dry and late dry period of Holstein cows. Mammary tissue expression of genes related to serotonin synthesis (*TPH1*), uptake (*SERT*) and signaling (*5-HTR5a*). Data are presented as ∆Ct in box and whisker plots displaying the median, first and third quartiles, minimum and maximum values with individual data points. Significance declared at (*) *P* ≤ 0.05, (**) *P* ≤ 0.001 (***) *P* ≤ 0.0001 and (#) denotes a statistical tendency at 0.05 < *P* ≤ 0.10.


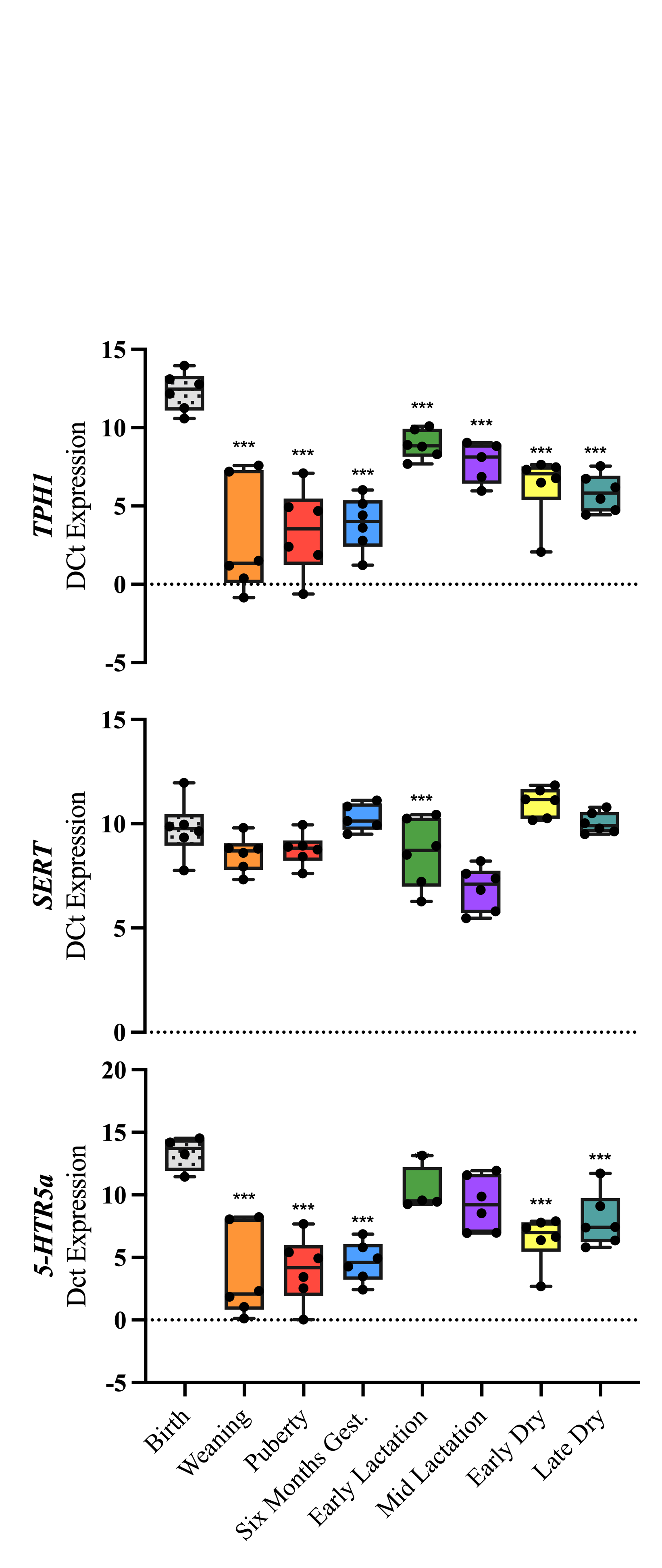

Supplement: S1 Figure — Mammary tissue expression of genes related to serotonin synthesis (TPH1), uptake (SERT) and signaling (5-HTR5a). Black dots represent individual data points. Bars are presented as LSM ± SEM. Significance declared at (*) P ≤ 0.05, (**) P ≤ 0.001 (***) P ≤ 0.0001 and (#) denotes a statistical tendency at 0.05 < P ≤ 0.10. (DOCX) [file pone.0319914.s002.docx]
